# A review of mechanisms and optimization strategies for clinical improvement after repetitive transcranial magnetic stimulation in bipolar disorder

**DOI:** 10.3389/fpsyt.2026.1849161

**Published:** 2026-07-02

**Authors:** Wojciech Korzeniowski, Piotr Koceniak, Napoleon Waszkiewicz, Krzysztof Walczewski, Gabriela Rusin, Jakub Antczak

**Affiliations:** 1 Doctoral School of Social Sciences, Jagiellonian University, Cracow, Poland; 2 Department of Education, Research and Development, Babinski Clinical Hospital, Cracow, Poland; 3 Centre for Brain Research, Jagiellonian University, Cracow, Poland; 4 Department of Psychiatry, Andrzej Frycz Modrzewski Krakow University, Cracow, Poland; 5 Department of Neurology, Jagiellonian University Medical College, Cracow, Poland; 6 Department of Psychiatry, Medical University of Białystok, Białystok, Poland

**Keywords:** bipolar disorder, cerebral cortex, connectivity, depression, EEG, mechanisms of action, repetitive transcranial magnetic stimulation, rTMS

## Abstract

**Introduction:**

Repetitive transcranial magnetic stimulation (rTMS) is an established therapeutic option in depression, especially in drug-resistant patients. While therapeutic efficacy in major depressive disorder (MDD) is confirmed in many meta-analyses, the results in patients with bipolar disorder (BD) are less unequivocal. This narrative review summarizes the current knowledge on possible mechanisms of action of rTMS in BD with focus on predictors of treatment response as well as on strategies that may improve therapeutic potential.

**Views:**

Available data suggest that a better clinical response may be associated with lower left frontal cortex volume, slower EEG activity, and lower default mode network activity. rTMS appears capable of inducing changes in transmitters and other neurochemical substances in BD but available studies are limited, compared with those in MDD. Furthermore, changes after rTMS include reduction in faster EEG bands and increase in blood flow, which were found mainly in the area of stimulation. Neuronavigation and connectivity guided targeting may improve therapeutic results of rTMS, which probably depend on connections of stimulated area with distant brain areas, especially regarding cognitive performance.

**Conclusions:**

Overall, several anatomical and neurophysiological factors were associated with therapeutic response. rTMS seems to induce significant neurochemical, connectivity, and EEG changes in BD but data in this area are still scarce. Navigated rTMS may improve clinical effects and provide better insights into therapeutic mechanisms, especially regarding cognitive performance of patients with BD.

## 1 Introduction

Bipolar disorder (BD) is one of the most frequent and severe psychiatric diseases. BD mainly comprises bipolar I disorder (BDI) and bipolar II disorder (BDII) subtypes. In BDI, the periods of depression, euthymia and mixed states alternate with manic episodes, whereas in BDII patients experience hypomanic rather than manic states. Other differences include periods of depression, which tend to be longer and more prevalent in BDII (1, 2). The lifetime prevalence of BD is 0.4 to 1.1% in high-income countries (3). The onset is typically in late adolescence or early adulthood (4). BD is one of the leading causes of psychiatric disability with annual costs (direct and indirect) of BDI being estimated at 202.1 billion dollars in the United States (5). BD profoundly affects quality of life, life expectancy and is also associated with somatic comorbidities, including diabetes, hypertension, hypothyroidism, and others (6). Up to 60% of individuals with BD attempt suicide during their lifetime, with mortality being estimated for 15%-20% (7).

The pathophysiology of BD partially overlaps with other psychiatric conditions, in particular with major depressive disorder (MDD). Genetic correlations have been documented between MDD and BDII as well as schizophrenia and BDI (8). Among the most extensively studied molecular alterations are polymorphisms of brain derived neurotrophic factor (BDNF) gene (9). Similar to MDD, BD is associated with inflammatory disturbances involving changes in levels of immune mediators such as IL-1, IL-6, and C-reactive protein. Other mechanisms associated with both MDD and BD include pathological microglial cell activation, dysregulation of the hypothalamic–pituitary–adrenal (HPA) axis, and an increased risk of metabolic disorders, e.g., diabetes (10, 11),. By contrast, dysregulation of calcium homeostasis with mutations in the CACNA1C calcium channel gene seems to be more specific for BD (12). Similarly, alterations in mitochondrial metabolism and mitochondrial DNA expression pertain to the core pathophysiology of BD but not MDD (13). The altered peripheral and cerebrospinal fluid levels of kynurenine and quinolinic acid, metabolites of an alternative pathway of tryptophan metabolism, have been reported in both disorders, yet the pattern of metabolite ratios differs significantly (14–16). Recently, increasing attention has been paid to the alterations in the gut microbiomes as a potential contributor to the development of BD and other psychiatric disorders (17, 18).

These genetic, cellular, metabolic, and endocrine changes are likely to contribute to changes in the brain structure and function, which can be investigated using neuroimaging and neurophysiological methods. Structural changes in BD include decreased grey matter volume (19), enlargement of lateral ventricles and white matter hyperintensities (20). Proton magnetic resonance spectroscopy (MRS) showed generalized reduction of N-acetylaspartate, elevated glutamate/glutamine levels, reduced creatine, increased choline ratio, and elevated lactate levels – findings that may reflect mitochondrial dysfunction (21, 22). MRS also documented reduced gamma-aminobutyric (GABA) levels in prefrontal cortex and cingulate areas (23, 24). Functional MRI studies using blood oxygen level dependent (BOLD) signal mainly reported reduced prefrontal activation and increased amygdala activation during emotional processing tasks, such as facial affect recognition (25–27). Cognitive tasks have been associated with stronger activation of the dorsolateral prefrontal cortex (DLPFC) and weaker activation of the ventrolateral prefrontal cortex (28, 29). During memory related tasks, hypoactivation has also been noted within insula (30). Studies on brain connectivity showed mainly increased default mode network (DMN) connections with bilateral precuneus, reduced connectivity with prefrontal cortex and middle temporal gyrus, as well as impaired DMN deactivation during task performance. In addition, dysregulation of fronto-limbic network involving prefrontal cortex and amygdala was found outside euthymic periods, whereas hypoactivation of the central executive network (CEN) has been observed in patients with cognitive deficits (31, 32).

Electroencephalographic (EEG) recordings in BD yielded heterogeneous results, with increases or decreases reported across different frequency bands in generalized or local way with dependence on the current phase (euthymic, depressive, or manic) and the presence of psychotic features (33–38).

Treatment of BD involves psycho- and pharmacotherapy, which despite decades of clinical experience and numerous available techniques and substances still remain suboptimal (39, 40). An alternative is the repetitive transcranial magnetic stimulation (rTMS), which is one of the most widely used modalities of brain stimulation, especially in treatment-resistant depression (41, 42). In rTMS trains of brief magnetic pulses are delivered to cerebral cortex by the coil held above the scalp. The magnetic field is able to induce electrical currents strong enough to depolarize local neuronal population. When applied over several weeks, stimulation may produce sustained changes in brain excitability and plasticity. If properly targeted, these changes may result in significant, therapeutic effect in a range of psychiatric and neurological conditions. rTMS pulses may be delivered with constant frequency, either high, i.e., ≥5Hz, or low i.e., ≤1Hz. An alternative way is the theta burst stimulation (TBS), which involves a more complex pattern comprising triplets of stimuli delivered at 50Hz and repeated at 5Hz. High frequency rTMS (HF-rTMS) and intermittent TBS (iTBS) are generally thought to induce plastic changes towards the long-term potentiation, whereas low frequency rTMS (LF-rTMS) and continuous TBS (cTBS) are believed to induce the long-term depression. TBS is thought to be related to neural oscillations within the brain. Although the TBS sessions are substantially shorter than conventional rTMS (typically three to nine minutes vs. up to 30 min) their neurophysiological effects are comparable, and their antidepressant efficacy has been shown to be noninferior (43, 44). The term “accelerated” rTMS or TBS, refers to therapeutic sessions which are administered more than once a day. Magnetic seizure therapy (MST) involves the use of high-frequency rTMS under anesthesia and assisted ventilation to induce the therapeutic seizure. Its efficacy seems to be comparable to electroconvulsive therapy (ECT) while the adverse effects may be milder (45).

In both BD and MDD, the most common cortical target for HF-rTMS and iTBS is the left DLPFC, whereas the right DLPFC is more often targeted with LF-rTMS and cTBS. This approach is based on the hypothesis of a prefrontal metabolic imbalance in depression, characterized by a relative hypoactivity of the left prefrontal cortex and hyperactivity of the right prefrontal cortex. However, this imbalance has been demonstrated primarily in MDD, and not in BD cohorts (46–48). rTMS is meanwhile a well-established therapeutic option in MDD, with FDA-clearance dating back to 2008. The antidepressant efficacy in MDD has been confirmed in multiple meta-analyses (49–52) with response and remission rates of 40-50% and 25-30% respectively (53). In BD, however, the results are less consistent. Some meta-analyses support the antidepressant effect (54–57), whereas others do not (58, 59). One clinically significant, albeit rare, side effect specific to BD is the induction of mania, which may limit the use of rTMS in this population (60). On the other hand, the most recent and one of the largest meta-analyses assessed the risk of induction of mania being no more frequent after real stimulation than after placebo. This meta-analysis confirmed also the therapeutic efficacy and efficiency of rTMS in BD (61). Furthermore, albeit not cleared for BD, rTMS was granted a breakthrough device designation by FDA in 2020 for this indication, which means that the evidence supporting this therapeutic option is strong enough to open an expedited path for FDA review. Despite this, current recommendations still mainly support use of rTMS limited to MDD (43).

At the same time, the body of research on mechanisms of action of rTMS in BD is growing rapidly. Available data include neurophysiological, laboratory, and functional neuroanatomical changes associated with rTMS as well as markers potentially related to favorable therapeutic response. While similar findings in MDD have already been summarized in numerous reviews and meta-analyses (62–68), no comparable work has focused on BD. In this review, we therefore aim to fill this gap by presenting radiological, laboratory and neurophysiological data on possible mechanisms of action of rTMS in BD. Furthermore, we intend to identify the factors that may contribute to its still limited and inconsistent efficacy and to highlight future research directions that could improve the clinical outcomes. Owing to heterogeneity of the available evidence, this review is conducted in a narrative manner.

## 2 Study selection

The PubMed and Scopus databases were searched independently by WK and PK for English-language original studies, reviews and meta-analyses published until March 2026. The search strategy combined the terms bipolar disorder and bipolar depression with terms referring to rTMS modalities and paradigms, including repetitive transcranial magnetic stimulation, rTMS, theta burst stimulation, TBS, magnetic seizure therapy, MST. These terms were cross-referenced with keywords related to radiological, anatomical, neurophysiological, endocrinological, laboratory, serological, and clinical methods and findings relevant to the mechanisms of bipolar disorder. During review of titles, abstracts and full texts, we excluded case reports and studies without rTMS treatment arm, and non-human studies, as well as studies not containing relevant data on mechanisms underpinning rTMS effect. Articles involving mixed populations were included only when the data for the BD subgroup were separately available or when the effect on BD could be inferred in other way. Effort was made to attain the good standards as defined by the Scale for the Assessment of Narrative Review (69).

**Figure 1:**
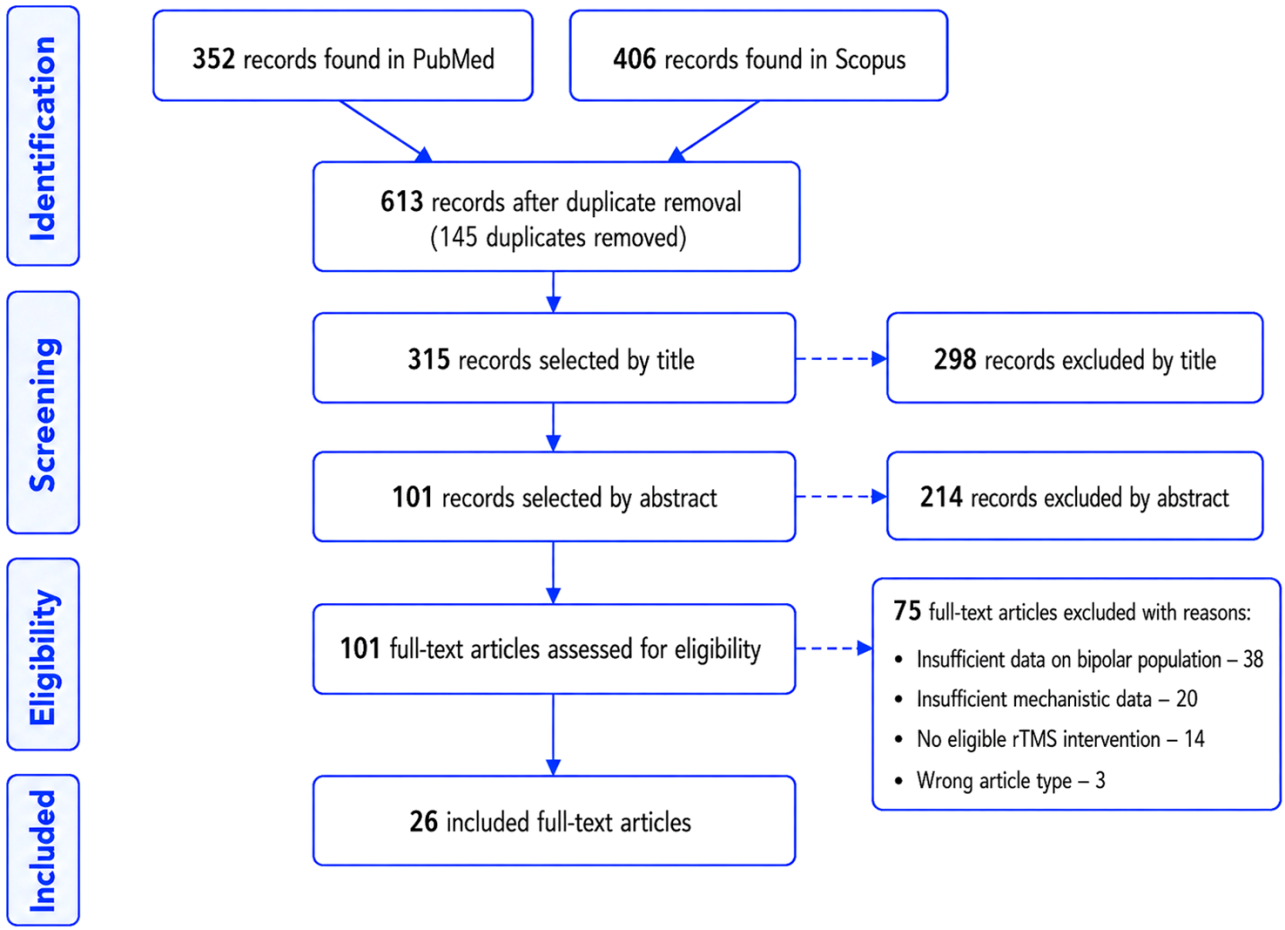
Article selection flowchart.

After duplicates were removed, the search yielded 613 records. After review, 26 articles were identified as eligible. 10 were randomized controlled trials, one was randomized trial including two active arms (no sham) and 13 were open trials. One study included data sets from randomized controlled trial and from open trial. Another one was a retrospective study (**Figure 1**, **Supplementary Table 1**).

### 2.1 Quality appraisal and risk of bias

To provide a more critical reading of the available evidence, we appraised the methodological quality and potential risk of bias of the studies discussed in this review. Randomized controlled trials were considered in relation to the main domains of the Cochrane Risk of Bias 2 framework, including the randomization process, deviations from intended interventions, missing outcome data, outcome measurement, and selective reporting (70). For non-randomized empirical studies, we used domains derived from the Newcastle–Ottawa Scale, focusing mainly on participant selection, comparability of groups or control of confounding, and the adequacy of outcome assessment (71). This appraisal was used to inform how much weight was given to individual findings in the narrative synthesis. Overall, the evidence base remains methodologically uneven. Only a small proportion of studies can be regarded as providing high-quality evidence, while vast majority fall into the moderate quality range. The most common limitations were small sample sizes, single-center or uncontrolled designs, limited use of sham-controlled comparisons, unclear or incomplete blinding procedures, heterogeneity of stimulation protocols, short follow-up periods, and inconsistent reporting of bipolar-specific outcomes.

## 3 Current evidence

### 3.1 Neurochemical findings predictive of rTMS effect and changes induced by rTMS

Knowledge about the relationship between rTMS and neurochemistry remains scarce. In a cohort of 37 patients, higher baseline levels of quinolinic acid, a neurotoxic metabolite, predicted response to right DLPFC cTBS (72). Another study found that 10 Hz rTMS over the left DLPFC increased medium- and long-chain fatty acid levels, which may exert immunomodulatory effects and influence neurotransmission (73). No studies investigated cytokines, other inflammatory markers, or HPA axis disturbances in relation to rTMS in BD.

### 3.2 Predictive value of structural and functional brain findings for therapeutic response to rTMS

Lower frontal cortical volume, reduced metabolic activity, and EEG slowing have been associated with better response to rTMS (74–77). In a study including 71 patients with MDD and 24 with BD, lower volume of the left superior frontal gyrus and left caudal middle frontal gyrus predicted response to 20 Hz rTMS over the left DLPFC (74). Although the BD subgroup was not analyzed separately, patients with BD showed greater improvement in MADRS scores than those with MDD, suggesting these findings might also apply to pure BD cohort. Notably, ACC morphology was not predictive of response, unlike in studies of pure MDD cohorts, where structural changes in ACC were consistently associated with treatment outcome (78, 79).

Responsiveness of BD to high-frequency stimulation and relation to metabolism were shown in the study of Speer et al. (80), in which 9 patients with BD and 13 with MDD received either 1 Hz or 20 Hz rTMS. Nearly half of the MDD group responded better to 1 Hz stimulation, whereas BD patients improved exclusively after 20 Hz stimulation. PET imaging showed that hypoperfusion across widespread brain regions predicted response to high frequency stimulation, while hyperperfusion predicted response to 1 Hz stimulation (in MDD group).

In another study, baseline EEG activity was evaluated in 13 patients with MDD and 8 with BD to predict response to 10 Hz rTMS over the left DLPFC. Lower alpha band power in bilateral parieto-temporal regions was associated with greater antidepressant response (75). Although separate analyses for MDD and BD were not performed, the scatterplots provided in the manuscript suggested a similar pattern of response in both groups. Consistent with these findings, in the study by Woźniak-Kwaśniewska et al. (76) BD non-responders had higher frontal and occipital alpha oscillations, whereas responders showed higher delta activity in the bilateral supramarginal gyri, inferior occipital lobes, and prefrontal cortex. Overall, both studies suggest that slower baseline brain activity predicts better response to rTMS. In the study by Kazemi et al. (77), 20 patients with BD received course of sequential bilateral DLPFC stimulation, consisting of high-frequency rTMS over the left DLPFC and low-frequency rTMS over the right DLPFC. Responders showed significantly lower default mode network (DMN) activity than both non-responders and healthy controls.

Two studies investigated the motor threshold (MT) in BD subjects undergoing rTMS. MT is one of measures of cortical excitability and is understood as a minimal strength of magnetic pulses elicited by TMS over motor cortex, capable to evoke motor potentials or visible muscle twitch. MT is estimated routinely at the beginning of therapy with rTMS to determine the strength of therapeutic magnetic pulses. It may be also estimated repetitively during the therapy to monitor for the possible need of adjustment of stimulation. In the study of Poleszczyk et al. (81), higher MT at baseline (and stronger therapeutic pulses) were associated with stronger t, whereas in the study of Pretalli et al. (82) it was the MT stability across the ten days of rTMS, which paralleled the better outcomes.

### 3.3 Structural and functional brain changes induced by rTMS

To date, no significant, structural brain changes following rTMS have been consistently demonstrated in BD. Metabolic, electrophysiological, and functional activity alterations have been observed more reliably near the stimulation site, and less consistently in the distant regions. The effects on brain connectivity were heterogenous. More pronounced effects were reported when rTMS was combined with other neuromodulatory techniques, such as transcranial direct current stimulation (tDCS).

In 16 patients with BD, iTBS over the left DLPFC did not produce significant morphometric brain changes at the group level (83). The only significant finding was a correlation between increased left hippocampal volume and improvement in nonverbal memory, although neither mood nor cognitive functions improved significantly overall. Another study applicating iTBS over the left DLPFC found increased GABA levels in the dorsomedial prefrontal cortex after active stimulation, again without corresponding clinical improvement (84). GABA is essential for inhibitory control of neuronal activity and its concentration was found to be low in BD with reactivity to pharmacotherapy (85).

Li et al. (86), in a mixed cohort of patients with BD and MDD, reported that a single rTMS session induced an immediate increase in BOLD signal both at the stimulation site (left DLPFC) and in limbic regions. Because only 147 stimuli at low frequency (1 Hz) were delivered over the left DLPFC, it can be assumed that metabolic response is readily elicited by rTMS. Kazemi et al. (87) examined EEG frequency band changes in 30 patients with BD. Fifteen of them received bilateral rTMS (10 Hz over the left DLPFC and 1 Hz over the right DLPFC), and remaining fifteen received unilateral, right-sided 1 Hz stimulation. Responders to bilateral stimulation showed significant reductions in alpha, beta, and gamma activity in medial and superior frontal regions and the cingulate gyrus. Responders to unilateral stimulation showed reduced gamma activity in parietal and occipital regions, with clinical improvement being smaller than after bilateral treatment. These results indicate, rTMS in BD patients can exert generalized attenuating influence on faster EEG activity. Similar, widespread EEG slowing was observed in three smaller studies, although they became apparent only with more advanced analyses, such as fractal dimension and graph theory–based connectivity methods (88–90).

Zhou et al. (91) investigated the effects of rTMS on brain connectivity in BD using occipital cortex stimulation with rTMS, tDCS, or their combination. Mood improved in both treatment arms with rTMS, but cognitive improvement occurred only after combined active tDCS and rTMS, alongside increased activity within the visual network. Similarly, fMRI studies showed increased activity in the opercular part of the inferior frontal gyrus after combined active tACS and rTMS over the left DLPFC, but not after rTMS alone (92). The opercular part of the inferior frontal gyrus is one of the central hubs of cognitive functions, in particular for language and speech processing (93). In another study, Wu et al. (94) investigated the role of the insula in therapeutic response. They found that 10 Hz rTMS over the left DLPFC reduced functional connectivity between the right anterior insula and right calcarine cortex in patients with BD, but not in those with MDD. In euthymic BD patients high frequency rTMS over the left DLPFC could transiently improve results in the MATRICS Consensus Cognitive Battery, which was associated with increased global functional connectivity density primarily in the prefrontal and frontal lobes, inferior temporal lobes, and bilateral parietal lobes (95). In other study a decrease in the connectivity within DMN was found along with improvement of mood and other symptoms (96). Noteworthy, described neurochemical, radiological and neurophysiological predictors of response to rTMS are paralleled by clinical predictors, summarized in the recent meta-analysis of Ventura et al. (61), which revealed that better response is associated with illness severity and increased number of sessions whereas longer disease duration possibly predicts less chance of achieving remission.

### 3.4 Connectivity guided rTMS

A series of experiments has examined the efficacy of rTMS with coil positioning guided by connectivity studies. This approach has gained increasing attention since the development of the Stanford Accelerated Intelligent Neuromodulation Therapy (SAINT) protocol. In this protocol, accelerated iTBS is applied to the site within the left DLPFC, which shows the strongest anticorrelation with subgenual ACC. The therapy comprises ten sessions per day during five consecutive days. SAINT was initially investigated in MDD (97) or in a predominantly MDD group (19 MDD and 2 BD) (98). Both trials showed a rapid and sustained improvement with one of them being stopped early for benefit. Subsequently, Li et al. (99) and Sheline et al. (100) reported significant improvement in MADRS scores in patients with BD treated with SAINT.

In another navigation-based study, combined 10Hz rTMS and tDCS applied over the left DLPFC showing the strongest connectivity with dorsal ACC improved the cognitive performance in BD patients (92). rTMS with concurrent tDCS was also applied to the left V1 brain regions functionally connected with the left DLPFC, resulting in mood and cognitive improvement (91). Visual cortex functionally linked to the orbitofrontal cortex was also targeted in the group of adolescent patients with BD with documented antidepressant efficacy (101). In other study, adolescents with BPII were stimulated over three sites, which were the hubs of CEN (F3, T5 and P3 EEG electrodes), likewise with improvement in cognition and mood (102). Finally, Wang et al. (103) compared efficacy of stimulation over the site within primary visual cortex functionally linked with DLPFC with stimulation of the site functionally linked with ACC. Only the latter was associated with the improvement in the Symbol Check Accuracy (one of several investigated cognitive tests). Notably, in contrast to most other cited studies, Wang et al. (92, 103) investigated patients in euthymic state. This design significantly reduced the confounding influence of antidepressant efficacy when investigating its effect on cognition.

We were unable to identify studies specifically referring to the mechanisms of action of MST in BD. [We found one study, which documented changes in brain excitability after six sessions of MST in a heterogeneous psychiatric cohort, which included some patients with BD, but no BD-specific mechanistic conclusions could be drawn (104)].

## 4 Future directions

The area which seems to be promising in treatment of affective disorders is the brain connectivity-based stimulation with examples cited above (91, 92, 102, 103). Such approach may expand the knowledge and therapeutic efficacy, especially with implementation of paradigm of the multiple site stimulation (102). The latter may be of particular importance in BD as the theory of imbalance between both prefrontal cortices, which determined in big part the neuromodulation strategy in MDD may less apply to BD and therefore stimulation over DLPFC alone may be insufficient. Another promising research direction, but less extensively studied is the use of multiple neurostimulation modalities as in the study of Zhou et al. (91). Future research may also include testing other stimulation frequencies and patterns in BD. This is warranted by the mentioned study in which none of BD patients responded to 1Hz rTMS, which was in contrast to the MDD group (80). Another cited study (77) also reported limited improvement after 1Hz over the right DLPFC. This indicates that the optimal pattern of TMS pulses may be distinctive from MDD and requires further investigation. Documented relation of EEG rhythms to therapeutic effect suggests neuromodulation, which takes into consideration the neural oscillations specific for BD may be more effective. An example is the paired associative stimulation, which according to our knowledge has not been investigated in BD. Finally, future studies searching for pathophysiologic markers of response to rTMS should take into consideration also clinical factors identified in the meta-analysis of Ventura et al. (61). Including both into future analyses may significantly increase understanding of rTMS mechanisms, in particular regarding causative potential of observed pathophysiological findings on therapeutic effect.

## 5 Conclusions

The efficacy of rTMS in BD is likely related to biological processes involved in BD pathophysiology as well as to morphological changes of cerebral cortex and to brain oscillations and connectivity. Findings that may be associated with better response include electroencephalographic slowing and lower DMN activity. rTMS also appears capable of inducing neurochemical, metabolic, and connectivity-related changes in BD, such as EEG slowing and increase of BOLD signal. These effects are observed mainly at the stimulation site, but often also in distant brain areas, as a result of activation of respective neuronal connections. Studies conducted to date have failed to demonstrate clear, structural brain changes induced by rTMS in BD. This contrasts with findings in MDD, where structural alterations have been reported in regions such as left hippocampus or left rostral ACC (79, 105, 106). Lack of detectable structural brain changes may reflect the decreased plasticity associated with BD, however such speculation requires support. Navigated rTMS may improve clinical outcomes and provide better insights into therapeutic mechanisms, especially regarding cognitive performance of patients with BD. No study, however, has directly compared the efficacy of navigated and non-navigated rTMS in BD, as it has been done in MDD (107).

Despite growing number of studies, there are still gaps in the current knowledge. In particular, we found no data on the relationship between the levels of cytokines and other markers of inflammation and rTMS efficacy in BD. This may be a notable limitation since inflammatory markers are known to predict the therapeutic response to ECT in both MDD and BD (108), and the levels of cytokines have been shown to change after rTMS in MDD (109). We also found no studies examining the effect of rTMS on mitochondrial and calcium metabolism, the HPA-axis function, or genetic factors in BD. There was a study investigating the association between BDNF polymorphism and response to rTMS, in the mixed population, but it included just five patients with BP among 31 patients with MDD (110). Furthermore, no study investigated potential mechanisms and other than clinical (longer stimulation period, higher stimulation strength (111)) predictors of treatment-emergent mania. Another significant gap is the absence of data on the effect of rTMS on frontal-vagal network and on efficacy of the cardiac-guided TMS, which is increasingly investigated in MDD patients (112, 113). Similarly, we could not find studies dealing with potential gender differences in response to rTMS among pure BD groups. One study on the mixed BD/MDD population revealed female sex to be associated with better response to rTMS (74), but this finding has not been later confirmed in the meta-analysis (61). Genders may react nonuniformly to rTMS since epidemiologic and symptomatic differences between women and men are well documented in BD (114, 115).

## Supplementary material

Supplementary Table 1 Characteristics and mechanistic findings of the studies included in the review. The table summarizes study design, participant characteristics, rTMS intervention parameters, clinical outcomes, investigated biological or neurophysiological measures, baseline predictors of treatment response, and biological changes observed during or after rTMS.
