# Supplementary material for: A review of mechanisms and optimization strategies for clinical improvement after repetitive transcranial magnetic stimulation in bipolar disorder
Source: Front Psychiatry. 2026 Jul 2;17:1849161. doi: 10.3389/fpsyt.2026.1849161 (PMC13372765; doi:10.3389/fpsyt.2026.1849161)
Supplement: Supplementary Table 1 — Characteristics and mechanistic findings of the studies included in the review. The table summarizes study design, participant characteristics, rTMS intervention parameters, clinical outcomes, investigated biological or neurophysiological measures, baseline predictors of treatment response, and biological changes observed during or after rTMS. [file Table1.docx]

| **Authors** | **Study type** | **Participants** | **Intervention** | **Clinical outcome** | **Biological function investigated** | **Relationship between baseline biological functions and effect of rTMS on psychopathology (treatment response)** | **Change of biological functions during rTMS** |  |
| --- | --- | --- | --- | --- | --- | --- | --- | --- |
|  | **Blood-derived neurochemical substances** | | | | | | | |
| Dellink et al., 2024 (72) | Randomized controlled trial | 37 BD (depressive phase), including 18 active and 19 sham | cTBS: 110% RMT, right DLPFC, 20 sessions | Depressive symptoms (HDRS): no significant change Mania symptoms (YMRS): no significant change Positive and negative syndrome: no significant change Psychomotor functioning: no significant change | Plasma level of tryptophan, kynurenine, kynurenic acid, and quinolinic acid | Higher quinolinic acid level predicted therapeutic response. |  |  |
| Yu et al., 2025 (73) | Uncontrolled pre-post study | 30 BD (depressive phase) | rTMS: 10 Hz, 110% RMT, 3000 pulses/session, left DLPFC, 14 sessions (successive days) | Depressive symptoms (HDRS): significant improvement  Anxiety (HAMA): significant improvement | Short- , medium- and long-chain fatty acids in plasma |  | Increase of the levels of various medium- and long-chain fatty acids. |  |
|  | **Imaging** | | | | | | | |
| Harika-Germaneau et al., 2022 (74) | Retrospective cohort study | 71 MDD, 24 BD (depressive phase) | rTMS: 20 Hz, 110% RMT, 3200 pulses/session, left DLPFC, 10 sessions | Depressive symptoms (MADRS): BD patients responded more frequently than MDD patients (58% vs 34%). | MRI: left DLPFC (with distinction of the superior frontal, caudal, and rostral middle frontal gyri), cortical volume and thickness, left and right hippocampus volumes, left and right amygdala volumes, and left and right ACC | Lower volume of the left hemisphere in superior frontal and caudal middle frontal regions in responders |  |  |
| Speer et al., 2009 (80) | Randomized controlled trial | 13 MDD, 9 BD (depressive phase); 1 or 20 Hz or sham | rTMS: 1 or 20 Hz, 100% RMT, 1600 pulses/session, left DLPFC, 10 sessions | Depressive symptoms (HDRS): BD patients improved exclusively after 20 Hz rTMS, while MDD patients responded to 1 Hz or to 20 Hz. Those who improved on one frequency tended to worsen on the other. | FDG-PET and/or [^15^O]H_2_O + MRI | • Baseline hyperperfusion (but not hypermetabolism) of bilateral dorsolateral and ventrolateral prefrontal cortex, medial and lateral temporal lobe, including the amygdala, parietal and occipital cortices, as well as thalamic, midbrain and cerebellar regions correlated with antidepressant response to 1 Hz rTMS • Baseline overall hypoperfusion predicted better response to 20 Hz rTMS. |  |  |
| Torres et al., 2023 (83) | Randomized controlled trial | 31 BD (depressive phase), including 16 active and 15 sham | iTBS: 120% RMT, 600 pulses/session, left DLPFC, 20 sessions | Cognitive functions (ISBD-BANC): no significant change | MRI: left, right and total hippocampus volume, total gray matter volumes |  | • Trend toward increase in left hippocampal volume in the treatment group. • Hippocampal volume increase associated with improved nonverbal memory functioning. |  |
| Diederichs et al., 2021 (84) | Randomized controlled trial | 17 BD (depressive phase) in predictive analysis, including 10 active and 7 sham; 12 in pre-post analysis, including 6 active and 6 sham | iTBS: 120% RMT, 600 pulses/session, left DLPFC, 20 sessions | Depressive symptoms: (HDRS, MADRS) lack of efficacy and overall low rates of clinical response in active and sham group (17% vs 16%) | MRS: GABA and Glx concentrations within medial prefrontal cortex |  | Increase of GABA in the medial prefrontal cortex after active stimulation, dissociated from antidepressant outcomes. |  |
| Li et al., 2004 (86) | Uncontrolled pre-post study | 6 MDD, 8 BD (depressive phase) | rTMS: 1 Hz, 100% RMT, 147 pulses, left DLPFC, 1 session | Not assessed | fMRI: regional BOLD activation, under the coil and in limbic regions. |  | • Increase in BOLD signal at the stimulation site (left DLPFC) and in the limbic regions such as: left hippocampus, bilateral thalamus (ipsilateral mediodorsal, bilateral pulvinar, anterior nucleus), bilateral putamen, bilateral parietal lobes and insula, right orbitofrontal cortex, left middle temporal cortex, and right prefrontal. • Significant deactivation during TMS compared with rest in the right ventromedial prefrontal cortex. |  |
| Zhou et al., 2024 (91) | Randomized controlled trial | 82 BD with cognitive decline, including 32 in active tDCS-active rTMS group, 27 in sham tDCS-active rTMS group, and 23 in active tDCS-sham rTMS group. | rTMS: 10 Hz, 110% RMT, 3000 pulses/session  tDCS: 20 min, hubs: 0.5-2 mA Stimulation target: primary visual cortex (region V1) functionally connected with DLPFC 15 sessions of rTMS/tDCS/both treatments | Depressive symptoms (HDRS): significant improvement in active tDCS-active rTMS group and in sham tDCS-active rTMS group Cognitive functions (THINC): significant improvement only in active tDCS-active rTMS group  Mania symptoms (YMRS): no significant change | fMRI: activity in functional brain networks related to emotional regulation and visual information |  | Combination of rTMS and tDCS increased activity in the calcarine sulcus and improved activity in visual network. |  |
| Wang et al., 2025 (92) | Randomized controlled trial | 66 BD (euthymic phase), including 18 in active tACS-active rTMS group, 17 in sham tACS-active rTMS group, 18 in active tACS-sham rTMS group and 13 sham tACS-sham rTMS. | rTMS: 10 Hz, 100% RMT, 3000 pulses/session tACS: 40 Hz, 30 min Stimulation target: highest structural connectivity point between left DLPFC and dACC 15 sessions of rTMS/tACS/both treatments | Cognitive functions (THINC): robust improvement in active tACS-active rTMS group compared to other groups; little, albeit significant improvement in active tACS-sham rTMS group compared to sham tACS-sham rTMS group. | fMRI: indicators of spontaneous activity in whole brain |  | Activation in opercular part of the inferior frontal gyrus in active tACS-active rTMS group. |  |
| Wang et al., 2023 (103) | Randomized controlled trial | 78 BD (euthymic phase), including 40 - stimulation of V1 region functionally connected with left DLPFC and 38 - stimulation of V1 region functionally connected with ACC; crossover design for each target (active→sham or sham→active) | rTMS: 10 Hz, 110% RMT, 3000 pulses/session, region of primary visual cortex (V1) functionally connected with left DLPFC/ACC, 10 sessions | Depressive symptoms (HDRS): no significant change Cognitive functions (THINC): significant improvement after active stimulation of V1 functionally connected with left ACC Mania symptoms (YMRS): no significant change | fMRI: functional connectivity between DLPFC or ACC and whole brain |  | No changes in functional connectivity. |  |
| Wu et al., 2024 (94) | Uncontrolled pre-post study | 10 MDD, 15 BD (depressive phase); data from final neuroimaging for 20 patients | rTMS: 10 Hz, 120% RMT, 1600 pulses/session, 12 sessions (5 per week during first 2 weeks, and 1 per week during next 2 weeks) | Depressive symptoms (HDRS): after treatment and during follow-up response rates ranged between 64 - 68%, while remission rates between 36 - 44%; rates were similar between BD and MDD patients. | fMRI: insula functional connectivity with other brain regions | In combined group, higher baseline functional connectivity between the right anterior insula and right superior temporal gyrus correlated with better antidepressant outcome. | Decrease in functional connectivity between the right anterior insula and the right calcarine cortex in BD group. |  |
| Wang et al. 2020 (95) | Uncontrolled pre-post study | 21 BD (euthymic phase) | rTMS: 10 Hz, 110% RMT, 1350 pulses/session, left DLPFC, 20 sessions | Depressive symptoms (HDRS): no significant change  Anxiety (HAMA): no significant change Cognitive functions (MCCB): significant short-term improvement  Mania symptoms (YMRS): no significant change | fMRI: gFCD |  | • Increase of gFCD in frontal lobes, inferior temporal lobes, and bilateral parietal lobes at the end of the second week of treatment.  • Increase of gFCD in frontal and temporal lobes compared to baseline at the end of the fourth week of treatment (changes smaller than after the second week). |  |
| Sheline et al. 2026 (96) | Randomized controlled trial for BD Uncontrolled pre-post study for MDD | 10 active MDD; 24 BD (depressive phase), including 12 active and 12 sham | iTBS: 90% RMT, 1800 pulses/session, region of left DLPFC most anticorrelated with subgenual ACC, 50 sessions (10 sessions per day) | Depressive symptoms (MADRS, BDI): significant improvement in combined group Insomnia (ISI): significant improvement in combined group Suicidality (SSI): significant improvement in combined group Ruminative thoughts (RTS): significant improvement only in BD after active stimulation | fMRI: connectivity within DMN |  | • Decrease in DMN connectivity in combined group. Similar change in MDD and BD. |  |
| Zhao et al. 2026 (101) | Randomized controlled trial | 52 adolescent and young adults BD (depressive phase), including 26 active and 26 sham | rTMS: 10 Hz, 100% RMT, 3000 pulses/session, region of the left V1 with the strongest connectivity with OFC, 15 sessions | Depressive symptoms (MADRS, HDRS, QIDS-SR): greater reduction in depressive symptoms after active rTMS, Anxiety (HAMA): no differences btween active and sham stimulation | fMRI: connectivity of V1 with other regions |  | • Active stimulation: Decrease in functional connectivity between V1 and the left anterior cingulate gyrus, increase between V1 and the right superior occipital gyrus• Sham stimulation: Increase in functional connectivity between V1 and the left anterior cingulate gyrus, decrease between V1 and the right superior occipital gyrus |  |
|  | **EEG** | | | | | | | |
| Micoulaud-Franchi et al., 2012 (75) | Uncontrolled pre-post study | 13 MDD, 8 BD (depressive phase) | rTMS: 10 Hz, 120% RMT, 2000 pulses/session, left DLPFC, 20 sessions | Depressive symptoms (BDI-SF): 57% of patients were responders. | EEG | Alpha band power in bilateral parieto-temporal regions negatively correlated with treatment response. |  |  |
| Woźniak-Kwaśniewska et al., 2015 (76); further re-use dataset in: Lebiecka et al., 2018 (88), Zuchowicz et al., 2019 (89), Olejarczyk et al., 2020 (90) | Uncontrolled pre-post study | 8 MDD, 10 BD (depressive phase) | rTMS: 10 Hz, 120% RMT, 2000 pulses/session, left DLPFC, 20 sessions | Depressive symptoms (MARDS, BDI, CGI, YMRS): 60% of BD and 50% of MDD patients were responders. | EEG | • In BD non-responders, power of alpha oscillations was significantly higher in frontal and occipital lobes.  • In BD responders delta activity showed higher activation in the right and left supramarginal gyri, inferior occipital lobes, and in prefrontal cortex.  • Theta activity showed nearly the same pattern of activation as delta activity, but more spread.  • Beta power was similar between BD responders and non-responders. | • Woźniak-Kwaśniewska et al., 2015. - no changes in EEG spectral power  • Lebiecka et al. – lower fractal dimension in BD responders across both higher and lower frequency bands. • Zuchowicz et al. (EEG analysis using phase-locking value indices) – higher indices in theta and beta frequencies in BD responders. Increase in delta, theta, and gamma rhythms along with increasing number of sessions in BD responders.  • Olejarczyk et al. (EEG analysis using directed transfer function) – EEG activity (in particular beta and gamma power) increased in the frontal lobe, mainly in the left hemisphere in BD responders. |  |
| Kazemi et al., 2018 (77) | Uncontrolled pre-post study | 20 BD (depressive phase), 80 healthy controls | Each session consisted of: initially low-frequency rTMS: 1 Hz, 120% RMT, 1500 pulses, right DLPFC; and then high-frequency rTMS: 10 Hz, 100% RMT, 2250 pulses, left DLPFC; 20 sessions (6 per week) | Depressive symptoms (BDI): 55% of patients met response criteria, while 15% met remission criteria.  Cognitive functions (VFT, RAVLT, WCST): significant improvement was observed in executive functions, verbal memory, but there was no changes in selective attention and verbal fluency. | EEG | Lower activity of LMN in responders. | • Higher activity in MPN and lower activity in SMN in patients  • Lower activity in SMN in responders  • Higher activity in SMN, visual network, and visual perception network in non-responders |  |
| Kazemi et al., 2016 (87) | Randomized trial | 30 BD (depressive phase), 15 in unilateral stimulation group and 15 in bilateral stimulation group | • Bilateral stimulation group: 1 Hz, 120% RMT, 1500 pulses, right DLPFC; followed by 10 Hz, 100% RMT, 2250 pulses, left DLPFC; 20 sessions (6 per week) • Unilateral stimulation group : 1 Hz, 120% RMT, 2500 pulses, right DLPFC, 20 sessions (6 per week) | Depressive symptoms (BDI): proportion of responders in the bilateral stimulation group was significantly higher than that in the unilateral group (80% vs 47%). The remission rate was similar between groups (40%). Anxiety (BAI): similar improvement in both groups | EEG: activity in functional networks related to emotional regulation, memory and visual perception |  | • Bilateral group - decrease in the density of the alpha, beta, and gamma frequencies were observed in the medial frontal gyrus, inferior frontal lobe, ACC, and bilaterally cingulate gyrus  • Unilateral group - no significant EEG changes. • Responders from bilateral group - significant reduction of alpha, beta, and gamma frequencies in medial, superior and middle frontal gyri, ACC and cingulate gyrus.  • Responders from unilateral group - decrease of gamma power in postcentral gyrus, precuneus, superior and inferior parietal lobules, cuneus, and angular gyrus, supramarginal gyrus, and the superior temporal gyrus. |  |
|  | **MT** | | | | | | | |
| Pretalli et al. 2012 (82) | Uncontrolled pre-post study | 55 MDD, 20 BD (depressive phase) | rTMS: 10 Hz, 95% RMT, left DLPFC, 10 sessions | In combined group of MDD and BD,significant improvement in depressive symptoms (MADRS, HDRS, BDI), sleep disorders (HDRS, items 4,5,6), and psychomotor retardation (ERD). Anxiety (Tyrer scale for anxiety, STAIYA) improved significantly only in patients with constant MT. Depressive symptoms: non-significant tendency towards better outcomes in BD than MDD (response rate: 41.8% vs 60%; remission rate: 16.4% vs 35%). | MT | In combined group of MDD and BD, both patients with constant and patients with changing MT improved significantly in depressive symptoms, sleep disorders, and psychomotor retardation. Patients with constant MT during treatment improved more significantly in depressive symptoms than those with change in MT. Anxiety improved significantly only in patients with constant MT.  BD patients with constant MT during treatment improved more significantly in sleep disorders and anxiety than those with change in MT. | No significant change in MT for the whole group. MT was stable for 45% of patients, increased for 29% of patients, and decreased for 25% of patients. |  |
| Poleszczyk et al. 2018 (81) | Uncontrolled pre-post study | 10 MDD, 30 BD (depressive phase) | rTMS: 10 Hz, 120% RMT, left DLPFC, 20 sessions | Depressive symptoms (HDRS, BDI): 35% of patients were reponders, 30% were remitters, and 58% were partial responders. No significant difference in overall antidepressant effect between MDD and BD. | MT, cortical silent period | • Negative correlation between the baseline MT and antidepressant effect in combined group and in BD.  • Correlation between the baseline cortical silent period and antidepressant effect in combined group and in BD. | • No change in MT in combined group. • No change in cortical silent period in combined group. |  |
|  | **None** | | | | | | | |
| Luo et al. 2024 (102) | Randomized controlled trial | 42 adolescent BD (depressive phase), including 22 active and 20 sham | iTBS: 80% RMT, 600 pulses to left DLPFC + 600 pulses to left ITG + 600 pulses to left PPC /session, (2 sessions per day) 15 sessions | Depressive symptoms (HDRS) improvement in sham and active group with trend toward better outcome in the active group. Cognitive improvement (DNCAS) only in the active group | No function directly investigated. However, the complex paradigm of neurostimulation gives insight into the mechanisms undepinning clinical effect. |  |  |  |
| Li et al. 2024 (99) | Uncontrolled pre-post study | 10 BD (depressive phase) | iTBS: 90% RMT, 1800 pulses/session, region of left DLPFC most anticorrelated with subgenual ACC, 50 sessions (10 sessions per day) | 80% patients achieved remission of depressive symptoms (MADRS) at follow-up | No function directly investigated. However, the complex paradigm of neurostimulation gives insight into the mechanisms undepinning clinical effect. |  |  |  |
| Sheline et al. 2024 (100) | Randomized controlled trial | 24 BD (depressive phase), including 12 active and 12 sham | iTBS: 90% RMT, 1800 pulses/session, region of left DLPFC most anticorrelated with subgenual ACC, 50 sessions (10 sessions per day) | 50% patients achieved remission of depressive symptoms (MADRS) after active stimulation and none after sham. | No function directly investigated. However, the complex paradigm of neurostimulation gives insight into the mechanisms undepinning clinical effect. |  |  |  |

ACC - anterior cingulate cortex, BAI Beck Anxiety Inventory, BD - bipolar disroder, cTBS stimulation - continuous theta burst, BDI - Beck Depression Inventory–II, BDI-SF - Beck Depression Inventory Short Form, BOLD - blood oxygen level dependent, CGI - Clinical global impression, dACC - dorsal anterior cingulate cortex, DLPFC - dorsolateral prefrontal cortex, DNCAS - Das-Naglieri Cognition Assessment System, ERD - Échelle de Ralentissement Dépressif (Depressive Retardation Rating Scale), FDG-PET - fluorodeoxyglucose-positron emission tomography, fMRI - functional magnetic resonance imaging, gFCD - global functional connectivity density, Glx - glutamate+glutamine, HAMA - Hamilton Anxiety Scale, HDRS - Hamilton Depression Rating Scale, ISBD-BANC - Bipolar Disorders–Battery for Assessment of Neurocognition, ISI - Insomnia Severity Index, iTBS - intermittent theta burst stimulation, ITG - Inferior temporal gyrus, LMN - language and memory network, MADRS - Montgomery–Åsberg Depression Rating Scale, MCCB - MATRICS Consensus Cognitive Battery, MDD - major depressive disorder, MPN - memory perception network, MRI - magnetic resonance imaging, MT motor- threshold, OFC - orbitofrontal cortex, PPC - Posterior parietal cortex, QIDS-SR - Quick Inventory of Depressive Symptomatology – Self-Report, RAVLT - Rey Auditory Verbal Learning Test, RMT - resting motor threshold, rTMS - repetitive transcranial magnetic stimulation, RTS - Ruminative Thought Scale, sgACC - sub genual anterior cingulate cortex, SMN - sensorimotor network, SSI - Scale for Suicidal Ideation, STAIYA - State Trait Anxiety, tACS - transcranial alternating current stimulation, THINC - THINC‐integrated tool, V1 - primary visual cortex, VFT - Verbal Fluency Test, WCST - Stroop Test, and Wisconsin Card Sorting Test, YMRS - Young Mania Rating Scale
